# Antibody Responses to *Sarcoptes scabiei* Apolipoprotein in a Porcine Model: Relevance to Immunodiagnosis of Recent Infection

**DOI:** 10.1371/journal.pone.0065354

**Published:** 2013-06-06

**Authors:** Melanie Rampton, Shelley F. Walton, Deborah C. Holt, Cielo Pasay, Andrew Kelly, Bart J. Currie, James S. McCarthy, Kate E. Mounsey

**Affiliations:** 1 School of Health and Sport Sciences, University of the Sunshine Coast, Maroochydore, Queensland, Australia; 2 Infectious Diseases Division, Queensland Institute of Medical Research, Herston, Queensland, Australia; 3 Global and Tropical Health Division, Menzies School of Health Research, Charles Darwin University, Northern Territory, Australia; 4 Centre for Advanced Animal Science, Department of Agriculture, Forestry and Fisheries, University of Queensland, Gatton, Queensland, Australia; 5 School of Medicine, University of Queensland, Herston, Queensland, Australia; Universidade Federal de Minas Gerais, Brazil

## Abstract

No commercial immunodiagnostic tests for human scabies are currently available, and existing animal tests are not sufficiently sensitive. The recombinant *Sarcoptes scabiei* apolipoprotein antigen Sar s 14.3 is a promising immunodiagnostic, eliciting high levels of IgE and IgG in infected people. Limited data are available regarding the temporal development of antibodies to Sar s 14.3, an issue of relevance in terms of immunodiagnosis. We utilised a porcine model to prospectively compare specific antibody responses to a primary infestation by ELISA, to Sar s 14.3 and to *S. scabiei* whole mite antigen extract (WMA). Differences in the antibody profile between antigens were apparent, with Sar s 14.3 responses detected earlier, and declining significantly after peak infestation compared to WMA. Both antigens resulted in >90% diagnostic sensitivity from weeks 8–16 post infestation. These data provide important information on the temporal development of humoral immune responses in scabies and further supports the development of recombinant antigen based immunodiagnostic tests for recent scabies infestations.

## Introduction

The ectoparasitic mite *Sarcoptes scabiei* causes a skin disease referred to as scabies in humans and as sarcoptic mange in other animal species. *S. scabiei* affects a wide range of animals worldwide, with particularly vulnerable animals including pigs, dogs, camelid species, wombats and koalas [1]. Scabies not only causes morbidity due to the severe, persistent itch, but may also lead to secondary infections, which can cause serious health complications [2], [3]. In animals, sarcoptic mange is associated with adverse welfare and reproductive outcomes. For example, mange infestation is linked to decreased testes mass and reduced reproductive capacity in male Iberian ibex (*Capra pyrnaica*) [4], and both male and female coyotes [5]. Mite antigens elicit inflammatory and allergic-type reactions in the host that vary in clinical presentation. In humans, ordinary scabies is characterised by a low mite burden (<15 mites in total), intense pruritus, papular and vesicular lesions at the site of infestation. In contrast, human crusted scabies is a rare, debilitating manifestation, that entails the formation of hyperkeratotic skin crusts containing an extremely high mite burden (>1000 mites/g skin) [6]. In wild animals measuring the extent of damaged skin may be useful proxy to evaluate mite burden [7]. Crusted scabies can be associated with immunosuppressive conditions, such as HIV or HTLV-1 infection, following organ transplantation, or induced by immunosuppressive drugs including corticosteroids [8]. Importantly however, it is also observed in a significant number of individuals with no overt immunosuppression or risk factor [9]. The immunopathology of scabies, particularly crusted scabies, remains poorly understood [10]. Host responses to *S. scabiei* are complex, with host species, previous exposure, mite immunomodulation, individual susceptibility and sex, all playing a likely role [11], [12], [13].

There are few methods to diagnose scabies that are sufficiently sensitive, cost effective and convenient. The “gold standard” for diagnosis is the identification of mites, eggs, or faeces from scrapings of infested skin or by identification of mite burrows. Despite 100% specificity, this method can have low sensitivity (<50%) [14], as it relies on physically locating mites on the host, problematic when mites are low in number. Clinical diagnosis can be hindered by atypical manifestations, or symptoms that mimic other skin conditions such as allergic dermatitis, or insect bites. Thus, specificity of clinical diagnosis may lead to problems with under or over diagnosis and inappropriate treatment of patients. Alternate diagnostic methods for scabies include polymerase chain reaction (PCR) [15], and dermoscopy [16], [17], but these still rely on the ability to locate mites or mite DNA. These methods are also not easily applied to clinical or public health settings.

The development of a reliable serologic method for diagnosis of scabies, such as enzyme linked immunosorbent assay (ELISA), would facilitate the control of scabies at both the individual and community level. There is no commercially available ELISA for the diagnosis of scabies in humans. ELISAs previously developed for the detection of mange in animals utilise whole mite antigen (WMA) extracts sourced from heavily infested animals, such as *S. scabiei* var. *vulpes* sourced from foxes [18], [19]. However, the reported specificity and sensitivity of these tests is variable [20], [21]. Although variants of *S. scabiei* are morphologically similar [22], they are predominantly host specific [23], [24]. Cross reacting proteins between different host variants have been identified via immunoblotting [25], but there is usually insufficient cross reactivity between host associated populations to use animal derived WMA extracts for diagnosis of human scabies for example [26], [27], [28]. Sourcing a *S. scabiei* var. *hominis* WMA extract for use in a human scabies diagnostic ELISA is not feasible due to the inability to undertake *in vitro* culture, and the low mite numbers in most human infections. Therefore, development of an alternative approach utilising *S. scabiei* recombinant antigens would be of benefit.

In previous studies several recombinant proteins with immunogenic potential have been identified [29], [30], [31]. Of these, the recombinant apolipoprotein Sar s 14 has emerged as a promising immunodiagnostic candidate. A 400 amino acid region at the C-terminus of this protein (Sar s 14.3), has been shown to elicit robust IgG and IgE responses in patients with ordinary and crusted scabies [32]. Recently, a quantitative IgE DELFIA using Sar s 14.3 showed 100% sensitivity in differentiating current scabies infection from non-infected and previously exposed patients [33]. A limitation in the development and optimisation of serological tests for scabies is the difficulty in rigorously defining the antibody response at different stages of infestation, and accounting for individual and host variability. Soluble extracts of *S. scabiei* have been found to modulate expression of several cytokines and lymphocyte surface molecules, which may result in down-regulated antibody responses during infestation [34]. Thus, it has not been determined at what point of infestation antibody responses to recombinant antigens such as Sar s 14.3 can be detected, or for how long antibody responses remain positive. In primary scabies infestation, the incubation period can be up to 6 weeks [21], with the patient often unaware of infestation during this period. This is of paramount importance in the clinical setting, as earlier sensitive detection would facilitate improved treatment and control by reducing transmission.

A tractable animal model of scabies has recently been utilised in pigs [35]. The clinical manifestations and disease progression in *S. scabiei* infested pigs closely resembles that of humans [36], including the development of acute and chronic mange, akin to ordinary and crusted scabies [37]. Thus our porcine model may be useful in the testing and comparison of different immunodiagnostic assays, providing leads to their use in both medical and veterinary settings.

The aim of this study was to monitor the progression of the porcine antibody response to scabies by measuring parasite-specific immunoglobulin levels (IgG, IgG1, IgG2 IgA and IgM), and to assess the suitability of the recombinant antigen Sar s 14.3 for diagnosis of porcine mange. We compared the diagnostic sensitivity of the recombinant antigen Sar s 14.3 to *S. scabiei* WMA extract, the current immunodiagnostic of choice for mange in animals. Moreover, we explored potential correlations between mange lesion severity and antibody responses. Dexamethasone was used in this study to enhance infestation intensity; producing clinical phenotypes akin to immunosuppression induced crusted scabies [35]. This allowed us to study the effect of corticosteroids on antibody responses in the presence and absence of mange. These groups were compared with pigs infested with mites but not treated with corticosteroids, which were expected to develop different clinical phenotypes depending on individual susceptibility.

## Materials and Methods

### Ethics Statement

Approval was obtained from the animal ethics committees of the Queensland Institute of Medical Research (Approval number 1266) and the Queensland Department of Agriculture, Forestry and Fisheries (Approval number SA 2009/07/294).

### Animal Trial

This study was undertaken as part of a larger project exploring scabies immunopathology in a porcine model, and was conducted from 10^th^ September 2009-March 2010. Twenty four three-week old female piglets (Large White variety) were obtained from 7 parent sows. They were early weaned and randomly allocated to one of four treatment groups (n = 6 per group). Group A: treated daily with 0.25 mg/kg oral Dexamethasone (Dex) and ears infected with approximately 2000 mites. Group B: ears infected with approximately 2000 mites. Group C: treated daily with 0.25 mg/kg Dex (Dex only control). Group D: No Dex or mite infection (negative control). Pigs were housed in four identical indoor rooms, with a 12 hour light/dark photoperiod and constant temperature of 24°C. Scabies infected and non-infected groups were housed separately, and non-infected pigs sampled first, to ensure that accidental transmission of mites did not occur. Dex treatments and experimental scabies infestation of the ears were carried according to protocols described previously [30], with *S. scabiei* infested skin crusts obtained from our existing mange pig model. The dose of 0.25 mg/kg dex was found to be sufficient to maintain mite infestations while minimising adverse effects [35]. After baseline samples were obtained, pigs commenced on Dex treatment for one week prior to mite infestation. The duration of the trial was 24 weeks. Skin lesions from each pig were scored weekly for clinical severity on a 1–8 scale (1 = mild papular rash (resembling pimples), 2–4 = papular rash, plus increasing exudates, red/inflamed skin, >4 = development of crusts, 8 = severe crusted mange, with development of lesions external to ears). Skin scrapings were collected from each pig fortnightly to approximate mite numbers, as described previously [35]. Approximately 10 mL of blood was collected from each pig fortnightly until week 12, then at weeks 16, 20 and 24 post-infestation. Serum was isolated by centrifugation at 3,000 g for 15 minutes, aliquoted and stored at −80°C until required.

### Source of Antigens

#### Whole Mite Antigen (WMA)

Heavily infested skin crusts were collected from mange infested pigs and *S. scabiei* var. *suis* isolated from skin using a dissecting needle under microscopy. Mites were stored in batches of 1,000–2,000 (approximately 10 mg) at −20°C until processing. To prepare antigen extracts, 100 µL of cold phosphate-buffered saline (PBS) was added to each tube, vortexed for 5 minutes and pelleted by centrifugation at 10,000×g for 5 minutes. Mites were washed with 100 µL of cold PBS with 1% sodium dodecyl sulfate (SDS) to remove adherent host materials, and then centrifuged at 10,000×g for 5 minutes. SDS residue was removed by washing twice with 100 µL of cold PBS. Mites were homogenised in 100 µL of cold PBS, and centrifuged at 10,000×g for 10 minutes. The homogenate was filtered in a 0.22 µm centrifugal filter unit (Millipore Ultrafree GV Durapore, Tullagreen) at 12,000×g for 3 minutes. Final protein concentration was determined by measuring absorbance at 280 nm using the Nanodrop ND-1000 spectrophotometer (Nanodrop Technologies, Wilmington, DE, USA). To determine whether the presence of small amounts of host IgG within WMA extracts could potentially confound results, we compared standard WMA extracts with extracts that had been subjected to IgG depletion (ProteoExtract Albumin/IgG removal kit, Merck Millipore, Kilsyth, Australia). As no significant differences were observed in serum binding between treated and untreated WMA extracts (students T-test p = 0.94, Figure S1), routine IgG depletion of WMA was not undertaken. The WMA preparations were stored at −20°C for later use.

#### Sar s 14.3

*S. scabiei* var. *hominis* Sar s 14.3 recombinant protein was expressed in *Escherichia coli* and purified as described previously [32]. Aliquots were stored at −20°C and protein concentration determined prior to use.

### Evaluation of Similarity of Sar s 14.3 between Human and Pig Derived *S. scabiei*

#### Sequence comparison

PCR was performed on *S. scabiei* var. *suis* cDNA derived from a pool of mites using primers specific to the *S. scabiei* var. *hominis* Sar s 14.3 fragment (F 5′TCGAATGTGAAACGAAACAATG 3′, R 5′GTGCAAATATTGTCTGATAGC 3′). The PCR reaction contained 1×PCR buffer, 0.4 µM of each primer, 5 units of high-fidelity Taq polymerase (Hot Star Taq, Qiagen, Doncaster, Australia), 2 µL template cDNA, and dH_2_0 to a final volume of 50 µL. PCR cycling conditions were: Initial activation 95°C, 5 min; 35 cycles of 94°C, 15 s 60°C, 1 min, 72°C, 1 min 30 s; with final extension of 72°C, 5 min. The PCR product was purified and sequenced. The resulting *S. scabiei* var. *suis* Sar s 14.3 sequence was compared to the corresponding *S. scabiei* var. *hominis* derived sequence (AF462196) by sequence alignment using ClustalW2 (http://www.ebi.ac.uk/Tools/msa/clustalw2/).

#### Western blot

To determine cross-reactivity of var. *hominis* Sar s 14.3 with mange infected pig sera, SDS PAGE was run with WMA (5 µg) and Sar s 14.3 (2 µg). Resolved protein bands were transferred to PVDF membranes, and blocked with 3% skim milk powder (SMP) in PBS overnight at 4°C. Membranes were incubated with sera collected from mange infected or non-infected pigs, diluted 1∶100 in 1% SMP-PBS, followed by goat-anti pig IgG-AP secondary antibody, diluted 1∶2000 (Abcam) using standard western blotting methods [32]. Membranes were developed and visualised using the phosphatase substrate BCIP/NBT (Sigma-Aldrich).

### Detection of Scabies Specific Antibodies by ELISA

Polystyrene plates (Greiner Bio-one) were coated at 4°C overnight with either WMA diluted in carbonate buffer (0.1M Na_2_CO_3_, pH 9.6) or Sar s 14.3 diluted in urea buffer (3M urea, 100 mM NaH_2_PO_4_, 10 mM Tris, pH 8.2), both at a concentration of 0.5 µg per well. Plates were washed twice with PBS with 0.5% Tween-20 (PBS-T) and blocked with 200 µL 1% Bovine Serum Albumin (HyClone, Utah) in PBS-T, incubated at 4°C overnight or 37°C for 2 hours, followed by washing twice with PBS-T.

Test sera were diluted in PBS-T containing 0.8M NaCl, with optimal dilutions for each antibody combination determined by checkerboard titration. For WMA, sera were diluted 1/1000 for detection of IgG, 1/100 for IgG1, IgG2 and IgM, and 1/20 for IgA. For Sar s 14.3, sera were diluted 1/100 for detection of IgG, IgG1 and IgM, 1/50 for IgG2, and 1/20 for IgA. Sera was aliquoted in duplicate at 50 µL per well. To evaluate ELISA performance and account for inter-assay variation, positive control serum, diluted at the relevant test concentration, was included on each plate. This control was derived from a pool of infected pigs, previously shown to elicit strong reactivity in western blot and ELISA. Additional controls included background (all antibodies replaced with PBS), omission of primary antibody, and omission of antigen. Plates were incubated at 37°C for 2 hours, and washed five times with PBS-T.

Secondary antibodies used were: Rabbit anti-pig polyclonal IgG-HRP (Sigma-Aldrich, 1/20,000); Mouse anti-pig monoclonal IgG1 and IgG2 (Thermo Scientific, Rockford, 1/100 or 1/500); Goat anti-pig IgA-HRP (Bethyl Laboratories, Montgomery, 1/2000); Goat anti-pig IgM-HRP (Bethyl Laboratories, 1/2000). As there is no porcine-specific IgE antibody commercially available, IgE reactivity was not evaluated. Secondary antibodies were diluted, incubated and washed as above. For detection of IgG1 and IgG2, a tertiary goat-anti mouse IgG-HRP antibody (Sigma, 1/500) was diluted, incubated, and washed as described above.

Substrate was prepared as per manufacturer’s instructions (Sigma-Fast OPD, Sigma-Aldrich) and 200 µL added to each well. Plates were developed in the dark for 30 minutes and read at 450 nm. To calculate optical density (OD), mean background values were subtracted from test samples and duplicates averaged. Corrected OD values were normalised by dividing by the positive control OD to obtain a standardised ELISA unit.

### Statistical Analysis

All analysis was carried out using GraphPad Prism version 5.0 (GraphPad Software, Inc.). Significance of differences between groups were assessed by repeated measures two-way analysis of variance (ANOVA), with Bonferroni post-tests to compare groups at each time-point. As this test requires sample groups to be matched (i.e. equal numbers across all time points), the first two weeks were not tested due to missing or insufficient sera samples for some pigs at these time points.

As a measure of potential diagnostic performance, sensitivity and specificity was calculated on all ELISAs, at all time points. Cut-offs were calculated at each week by taking the average of the negative control group (group D) +2 Standard Deviations. Sensitivity percentages were calculated using the following formula: ELISA positive/scabies positive pigs × 100, whereas specificity percentages were calculated by: ELISA negative/scabies negative pigs × 100. Overall ELISA performance was also compared using Receiver Operator Characteristic (ROC) curve analysis, which provides detailed information about discrimination between positive and negative samples, allowing determination of optimal cut-offs by plotting assay sensitivity versus specificity across a range of cut-off points. The ‘optimum’ is the cut-off where the highest sensitivity and specificity is achieved. For this, IgG, IgG1 and IgG2 assays were compared for WMA and Sar s 14, using ELISA values obtained during peak of clinical infection (weeks 8–20).

To assess if clinical phenotype was correlated with antibody response, Spearman correlation coefficients were calculated between the skin lesion scores and ELISA values from scabies infected pigs over the peak of clinical infection (weeks 8–20). Due to the possible confounding influence of Dex immunosuppression on antibody response, pigs in the non-Dex treatment group were assessed independently.

## Results

### Similarity and Cross Reactivity between *S. scabiei* var. *suis* and var. *hominis* Sar s 14.3

**Figure 1:**
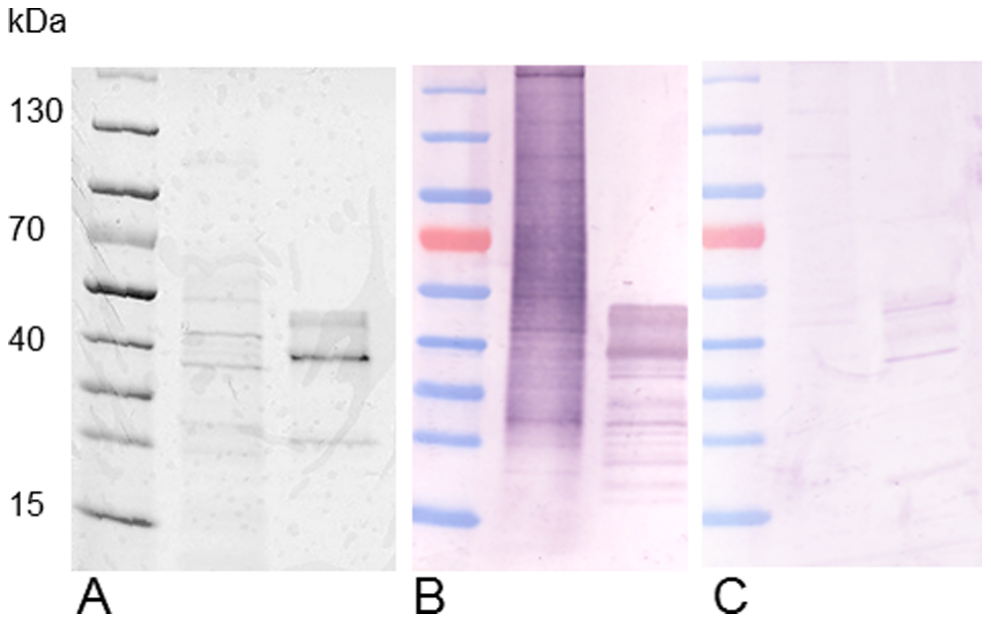
IgG reactivity of S. scabiei whole mite antigen extract (WMA) and Sar s 14.3 with porcine sera. A: 4–20% coomassie stained SDS-PAGE gel loaded with lane 1: prestained molecular weight marker, lane 2: WMA, 5 µg, lane 3: Sar s 14.3 (2 µg). Corresponding Western Blots probed with sera from mange infected pigs (B) and non-infected pigs (C).

Sar s 14.3 cDNA was PCR amplified from *S. scabiei* var. *suis* using primers based on the *S. scabiei* var. *hominis* sequence. The sequenced pig mite PCR product had 100% amino acid identity to the human mite Sar s 14.3, and was submitted to Genbank (Accession KC691249). Recombinant Sar s 14.3 derived from *S. scabiei* var. *hominis* was recognised by sera from mange infected pigs, with strong IgG binding to both Sar s 14.3 and the WMA positive control (Fig. 1B). Non-infected pig sera reacted only weakly with these antigens (Fig. 1C).

### Clinical Progression of Mange Infection

**Figure 2:**
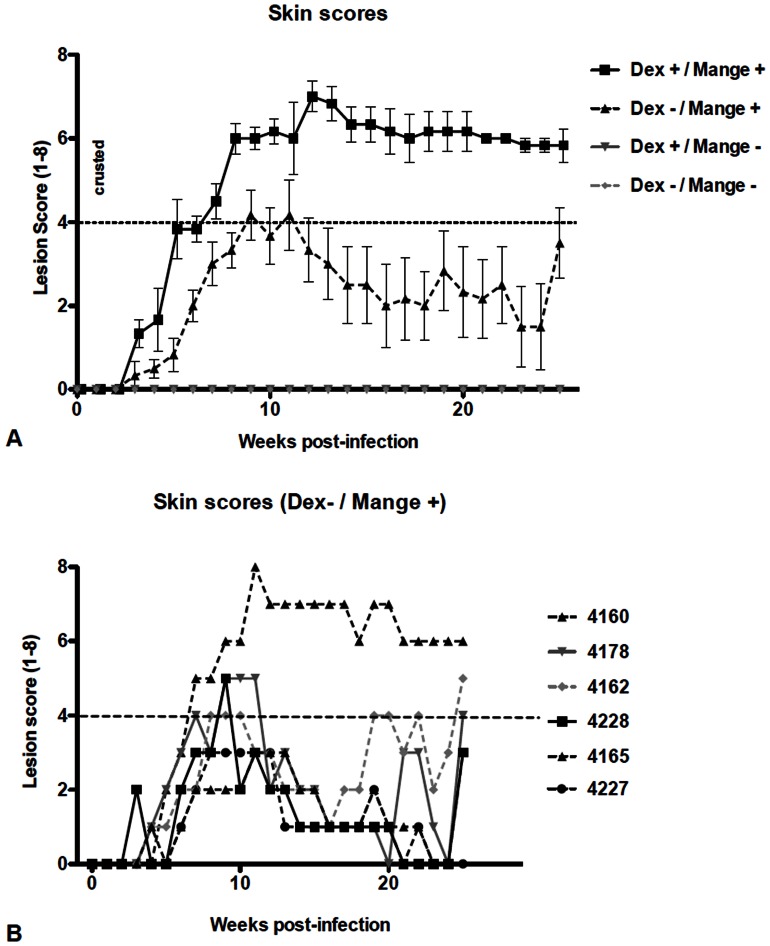
Lesion development in mange infected pigs. Ear lesions were scored weekly. Score of 1–4: acute mange with generalised rash and papular lesions of increasing density, >4: development of increasing encrustment, 8: extensive encrustment spreading external to ears. A: Comparison of treatment groups (n = 6 per group), error bars represent mean ±SEM. B: Clinical variation in mange severity in individual pigs from the non-Dex treatment group.

A range of clinical manifestations were observed during the 24 week animal trial. Lesions were first detected in infected pigs at 3–4 weeks post infection. Dexamethasone treatment accelerated lesion development and severity, with crusted mange (lesion scores 4–8) observed in all Dex treated pigs from weeks 8 to 24 (Fig. 2A). One pig in this group was euthanized at week 20 when clinical severity reached AEC welfare criteria. Mange positive pigs not receiving Dex treatment showed a range of clinical manifestations, including acute and crusted mange (Fig. 2B). Most pigs in this group showed a peak in lesion severity between weeks 8 and 12, before a reduction in symptoms occurred from weeks 12–16, and stabilising from weeks 16–24. Two pigs in this group developed crusted mange despite the absence of corticosteroids. Pigs in the non-infested groups did not develop skin lesions, nor were mites recovered in skin scrapings at any time during the trial.

### Development of Scabies-specific Antibodies during Infection

#### Antibody Responses to Whole Mite Antigen (WMA)

**Figure 3:**
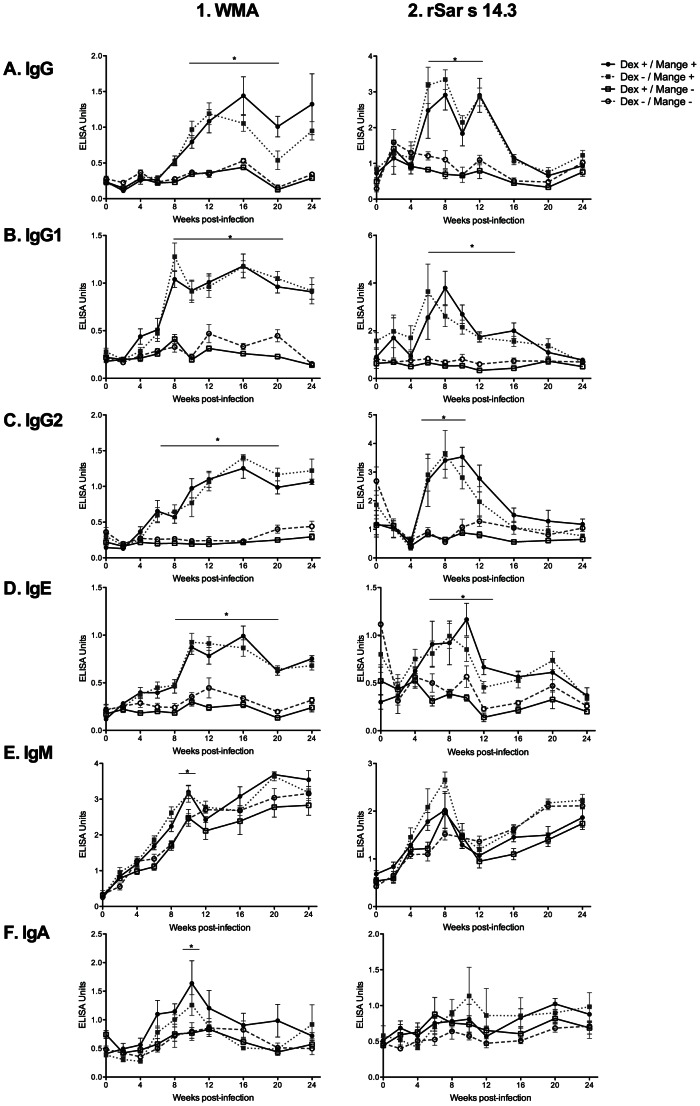
Isotype specific WMA (1) and Sar s 14.3 (2) antibody responses over 24 weeks for the four pig treatment groups. Error bars represent mean ±SEM. *: p<0.05, compared to group D (Dex −/Mange -) (two way repeated measures ANOVA). The first two weeks were not tested by ANOVA due to the requirement for matched numbers, and insufficient sera samples from some pigs.

Pigs from both mange positive groups developed robust IgG, IgG1 and IgG2 responses to WMA extract. Statistically significant differences in specific IgG were evident in mange positive pigs compared with non-infected pigs from week 10 post infection and continued to week 24. Responses in IgG1 and IgG2 subclasses were similar to IgG, but occurred slightly earlier, at week 8 (IgG1) and week 6 (IgG2) post-infection (Figure 3-1, A–C). Levels of IgM increased in all groups over the course of infection. At week 10 however, mange positive pigs had significantly increased levels IgM (Fig. 3-1, D). In the mange positive pigs there was a trend toward increased IgA levels, but this also only reached statistical significance at week 10 (Fig. 3-1, E).

#### Antibody responses to Sar s 14.3

Significantly increased IgG, IgG1 and IgG2 responses were observed to Sar s 14.3 in mange positive pigs from weeks 6–12 post infection compared to non-infected pigs (Fig. 3-2, A–C). In contrast to WMA, there was a decline in IgG antibody levels to Sar s 14.3 occurring from around weeks 12–16, although mange positive pigs could still be differentiated from non-infected pigs. Like WMA, there were increases in IgM against Sar s 14.3 in all groups, but no significant differences between groups (Fig. 3-2, D). There were no significant differences in the IgA response to Sar s 14.3 between treatment groups, although a trend for increased IgA levels in the mange positive groups could be observed (Fig. 3-2, E).

### Effect of Dexamethasone Treatment on Antibody Response

For some isotypes there appeared to be a trend towards a slightly lower antibody response in the Dex positive, mange negative pigs, but there were no statistically significant differences in antibody responses in the Dex treated groups either in the mange positive or non-infected groups (Fig. 3).

### Correlation between Lesion Severity and Antibody Levels in Mange Infected Pigs

**Figure 4:**
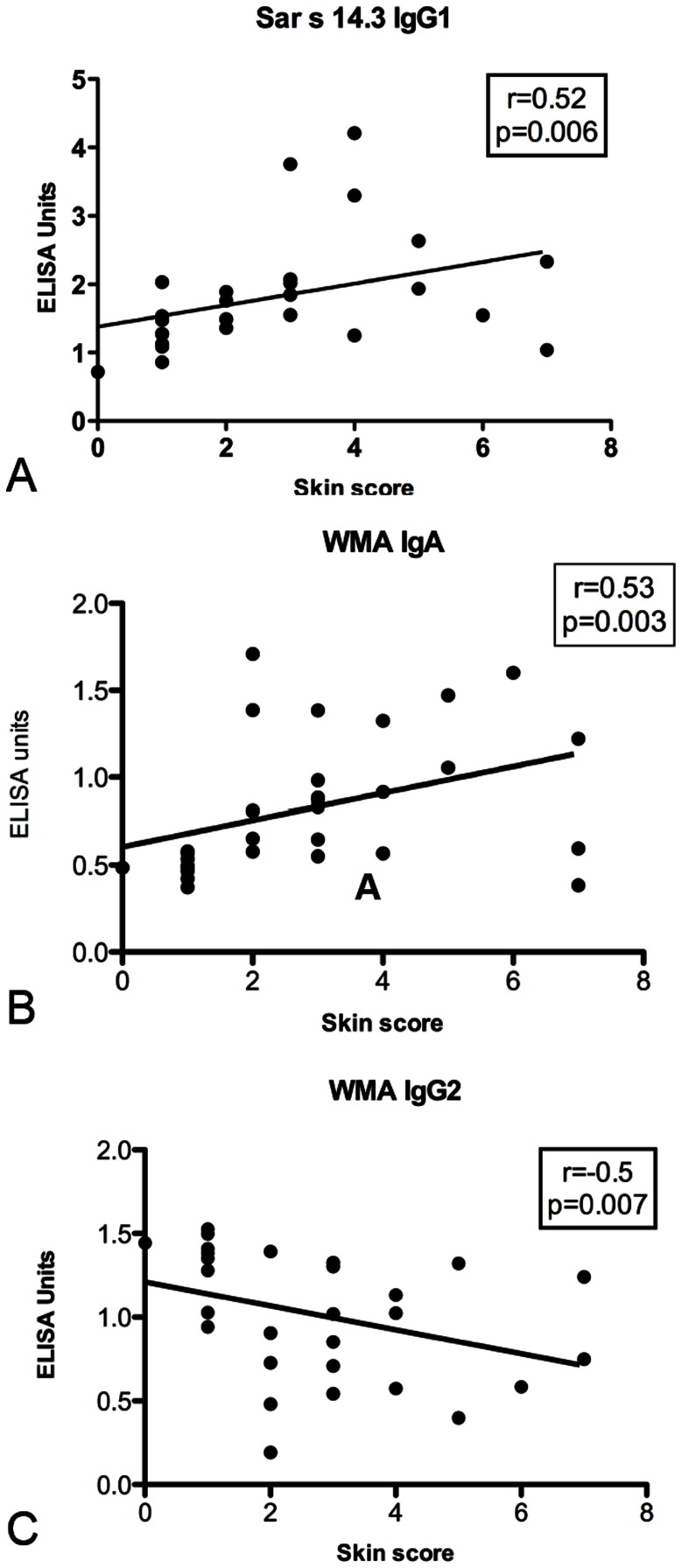
Correlation between lesion severity and antibody response. Scatter plot showing lesion score and ELISA units from mange infected pigs in the non-Dex treatment group from weeks 8–20 post infection. Each point represents the lesion score and antibody level for an individual pig at a single time point. R: Spearmans correlation coefficient.

Moderate positive correlations were observed in the anti-WMA, IgA (r = 0.53, p = 0.003) and anti-Sar s 14.3, IgG1 (r = 0.52, p = 0.006) ELISAs. Conversely, a negative correlation was observed in anti-WMA IgG2 ELISA (r = −0.5, p−0.007) (Fig. 4). There was no correlation observed for any other antigen/antibody combinations.

### Comparison of Diagnostic Sensitivity and Specificity of WMA and Sar s 14.3

Only total IgG and its subclasses were considered here, as IgM and IgA were not shown to be diagnostic in this study. Across peak clinical infection both antigens gave excellent diagnostic sensitivity and specificity (>90%). For WMA, 100% sensitivity was observed from week 8 with IgG and IgG1 and from week 12 with IgG2. Overall, the sensitivity of Sar s 14.3 ELISA was higher earlier in infection than WMA. For example, for Sar s 14.3 IgG1, 45% sensitivity was observed at week 2, and 83% at week 6. In contrast, WMA IgG1 sensitivity at week 6 was only 50% (Table 1). However, diagnostic sensitivity of Sar s 14.3 declined from week 16–20 post-infection. ROC analysis confirmed the good sensitivity achieved over the course of clinical infection (Table 2). Area under curve, an overall indicator of performance, was >90% for both antigens, but was higher for the WMA ELISAs. For both antigens, the IgG1 subclass gave the most sensitive detection, with 100% for WMA, and 97% for Sar s 14.3 (Table 1).

**Table 1:** Sensitivity and Specificity of ELISA tests for IgG to Whole Mite Antigen extract (WMA) and Sar s 14.3.

	IgG				IgG1				IgG2				
Week	WMA		Sar s 14.3		WMA		Sar s 14.3		WMA		Sar s 14.3		
	Sens (%)	Spec (%)	Sens (%)	Spec (%)	Sens (%)	Spec (%)	Sens (%)	Spec (%)	Sens (%)	Spec (%)	Sens (%)	Spec (%)	
0	0	100	0	100	0	100	0	100	0	100	0	100	
2	0	100	0	100	18	83	45	100	0	100	20	100	
4	0	100	0	100	25	100	25	100	8	100	18	100	
6	58	100	67	100	50	100	83	100	75	100	67	100	
8	100	100	100	100	100	92	100	100	92	100	100	92	
10	100	100	100	100	100	100	100	100	92	100	100	82	
12	100	100	92	92	100	80	100	100	100	92	50	100	
16	100	100	83	100	100	100	100	100	100	100	33	100	
20	83	100	64	100	100	100	58	100	100	100	33	100	
24	100	100	10	100	100	100	0	100	100	91	0	100	

**Table 2:** Receiver Operator Characteristic (ROC) comparison of sensitivity and specificity of ELISA tests for Whole Mite Antigen extract (WMA) and Sar s 14.3 from week 8 to 20 post-infestation.

	WMA			Sar s 14.3		
	IgG	IgG1	IgG2	IgG	IgG1	IgG2
**AUC** a	0.95	0.99	0.98	0.91	0.97	0.91
**S.E** b	0.019	0.004	0.012	0.025	0.015	0.027
**Cut-off**	0.43	0.49	0.45	0.93	1.02	0.95
**Sensitivity** c	90%	100%	95%	90%	97%	87%
**Specificity** c	90%	90%	90%	70%	92%	91%

a AUC- area under curve.

b S.E- standard error.

c Optimum sensitivity and specificity determined at cut-off indicated.

## Discussion

Diagnosis of scabies in humans and animals remains problematic. Smets & Vercruysse [38] conducted a large prevalence survey on mange in Belgium pig farms, and compared diagnostic techniques, with identification of mites considered as the “definitive diagnosis”. They found that clinical scoring of lesions (Average Dermatitis Score, ADS) was the most useful for diagnosis of mange in herds, but importantly, only 45% of ADS positive animals were positive for mites in ear scrapings. Such results highlight the present difficulty in diagnosing mange in individual animals.

An alternative diagnostic method for scabies may be immunodiagnostic ELISA utilising *S. scabiei* recombinant antigens, with the diagnostic potential of Sar s 14.3 recently demonstrated for human scabies [33]. The objective of this study was to utilise a porcine experimental model to compare Sar s 14.3 and *S. scabiei* var. *suis* WMA ELISA diagnostic sensitivity over the course of infection, and to investigate the effect of mange severity and corticosteroid immunosuppression on antibody response.

Clinical manifestations in the porcine trial proceeded as expected, with dexamethasone increasing infection severity and duration. In the non-Dex pigs, we observed a clinical peak at 8–10 weeks, before most pigs had a decline in clinical score. This temporal pattern of infection in pigs has been well documented [37], [39]. Two non-Dex pigs still developed crusted mange, indicative of differences in individual susceptibility, although it is important to note that these pigs were not siblings. The apparent recrudescence of symptoms at week 24 in some of the non-Dex pigs may represent reinfestation from heavily infested pigs, or alternatively, a cumulative immunomodulatory effect exerted by long term mite infestation causing a true clinical recrudescence. Mites have been shown to secrete molecules which modulate cytokine expression, and in particular Th1/Th2 balance [14], [34], [40], [41], [42], however the effect of this on humoral immunity or clinical outcome has not been clearly elucidated.

We confirmed a robust IgG response in pigs experiencing a primary mange-infestation to both WMA and Sar s 14.3. In previous studies the total IgG responses to *S. scabiei* WMA have been explored, but here we extend understanding by defining circulating IgG subclasses, IgM and IgA. Increased total IgG is commonly observed with scabies infection [9], [27]. In our study, levels of WMA specific IgG, IgG1 and IgG2 started to increase from weeks 6–8, and peak at week 12–16, before either declining slightly or plateauing. Other studies in pigs, dogs and goats show a similar progression, with antibody titres increasing slowly before plateau [13], [43], [44], [45], [46]. IgG1 and IgG2 responses to WMA were in accordance with that observed for total IgG, although elevation was evident slightly earlier.

Total IgG, IgG1 and IgG2 responses to Sar s 14.3 were also strong in infected pigs from weeks 6–12 post-infection. However, unlike WMA where seropositivity persisted or declined slightly, there was a marked decline in the response to Sar s 14.3 after week 12, and no significant differences between positive and negative pigs was apparent at weeks 20–24. The reasons for this decline are not immediately apparent. Age related factors might be possible, but immunocompetence and adult comparable IgG levels in pigs are reported to be achieved from weeks 8–10, after which total IgG is stable or increases until about 25 weeks [47], [48], [49], [50]. Few studies have looked at the effect of longer term mange infestations on humoral antibody responses. While it is tempting to speculate that this was associated with the decline in lesion severity, especially in the mange positive dex negative group, even pigs that remained heavily infested had a decline in IgG antibodies. It is possible that immunomodulation exerted by mites, or immunoresistance may cause a subsequent decrease in antibody levels [12], [13], [27], [34]. It has been shown that human subjects currently uninfected but with previous scabies exposure do not react to this antigen [32], and vaccination of rabbits with Sar s 14.3 failed to induce protective immunity in *S. scabiei* challenge trials [29]. Taken together, our results provide support that Sar s 14.3 reactivity is transient and not allow a protective immune response to scabies.

Another consideration of our study is that we used only female animals in this trial. Sarasa *et* *al* found marked reductions in antibody responses to both primary and secondary *S. scabiei* exposure in male ibex compared to females [13]. Sex related, hormonal linked, immunological differences have been observed in many other parasitic infections [51] with males often having increased susceptibility to disease. While it is uncertain whether this can be extrapolated to other animals (and humans) infested with sarcoptic mange, sex and exposure related differences in scabies ELISA diagnostic sensitivity should be evaluated in future studies.

As IgG subclasses diverged after speciation, “same name subclass” function cannot be extrapolated among mammals [52], so caution must be taken when comparing human and porcine IgG subclass data. Porcine IgG isotype bias has been described in relation to resistance and susceptibility to disease, with IgG1 associated with allergic type 2, and IgG2 with cell mediated type 1 immune responses [53]. Thus, in the context of Th1/Th2 directed responses, the IgG1:IgG2 ratio in pigs could be considered analogous to an IgG4:IgG1 ratio in humans. The development of crusted scabies in humans appears to be associated with a non-protective Th2 response [10]. Our data give some limited support to this hypothesis, as IgG1 levels in pigs were positively correlated with lesion severity, whereas IgG2 levels were inversely correlated.

A limitation to this study was that no porcine specific IgE antibody is commercially available, although some authors have shown that cross reactivity between human and porcine IgE exists [54] [55]. Several human studies report increased total and Sar s 14.3 specific IgE in scabies [9], [25], [32], and the development of a Sar s 14 ELISA for human scabies has focussed on detection of IgE, rather than the more commonly utilised IgG, which was considered a less specific marker of infection [32], [33]. In contrast, IgE responses to scabies in other animals are variable. A lack of IgE binding during primary infestation was observed in a significant proportion of *S. scabiei* infected dogs [27] and sheep [56], whereas in another study goats mounted a strong IgE responses to both primary and repeated mite challenge [46]. IgE responses also increased significantly in sheep during secondary infestation [56].From this it appears that differences may exist between the immune responses of humans and other animals to scabies, and between primary and secondary infestations. Thus, future ELISA design should consider the possibility of using different secondary antibodies for diagnosis in medical and veterinary settings.

Previous studies have reported conflicting results for scabies associated IgA development. IgA is usually more abundant in mucosal regions than in serum. Arlian [27], [57] and Hill [58] reported significant decreases in serum IgA in scabies infested subjects compared to controls. Increased levels of IgA have been found in scabietic lesions of pigs [59], so decreases in serum IgA may reflect migration to the site of infection. Interestingly however, Walton [32] showed significant increases in Sar s 14.3 specific IgA in sera from crusted scabies patients. Although conclusions made from this data are limited, there was some trend for increased WMA specific IgA in mange infected pigs, particularly those in the dex treated group where high lesion scores were observed. However this was only statistically significant at week 10. Similarly in the non-dex, mange infected pigs, a moderate, but statistically significant positive correlation between lesion score and WMA IgA level was observed.

IgM is traditionally considered the first line of the humoral immune response, and increased total IgM has been demonstrated in scabies patients [27], [57]. While we saw a trend for increased IgM in mange infected pigs, this was only significant at week 10 with WMA. The upward trend observed for all groups is likely non-specific, attributable to the low specificity and high avidity of IgM, combined with the development of the pig immune system which involves increasing total IgM [60]. The rate of IgM increase is especially rapid during the first eight weeks [49], [61].

At week 0, prior to experimental infection or treatment, high antibody responses were observed in some pigs to Sar s 14.3. Pre-trial testing showed that all parent sows of the pigs used in this study tested negative for Sar s 14.3 antibodies (Figure S2). Unlike humans, the placental barrier prevents immunoglobulins from passing into the developing piglet, although high levels of “pre-adaptive” IgG and IgA antibodies are obtained from colostrum [60]. Kuhn [31] also reported that non-infected porcine sera gave high reactivity to some recombinant *S. scabiei* proteins, and attributed this to the fact that the recombinant proteins were produced in *E. coli*, of which pigs have high levels of environmental exposure. Notably, some piglets in our trial were treated for an outbreak of scours (*E. coli*/diarrhoea) after room and pen allocation, but prior to Dex treatment or infection. This was most severe in the one of the two rooms allocated to mite negative groups, with most individual pigs in this room recording high baseline Sar s 14.3 ELISA ODs accordingly. Although the recombinant protein used in this study was purified with nickel affinity chromatography, these data may reflect a response to residual *E. coli* contaminants. Indeed, some minor binding was also observed to Sar s 14.3 in mange negative sera in the western blots. This issue could be resolved in future studies by more stringent purification, endotoxin testing and removal, or a different protein expression system. Pre-absorption with *E. coli* extracts did not successfully reduce background reactivity in previous experiments [20].

Understanding the impact of corticosteroids on antibody responses to scabies is of relevance, as crusted or atypical presentations of scabies can often occur in corticosteroid treated patients, and the ability of a scabies immunodiagnostic to correctly identify these cases is important. Although the effect of dexamethasone on the clinical development of mange was readily apparent in this study, we saw very little difference in antibody responses between mange infected, Dex treated and control groups. Pigs have been reported to be less susceptible to Dex treatment than other species. For example, Flaming [62] found that IgG antibody responses were unaffected in Dex treated pigs, despite receiving much higher doses (2–6 mg/kg) than used in our study (0.2 mg/kg). Similarly, Dex did not inhibit serum IgE levels in a mouse model of atopic dermatitis [63]. This is in contrast to cattle, where low doses of Dex (0.04 mg/kg) decreased antibody production [64].

### Implications for Diagnostics

Previous studies have shown Sar s 14 is expressed at high levels in scabies mites, is immunogenic, but has minimal cross-reactivity to house dust mite specific antibodies [29], [32], [33]. It is presumed that all host variants of *S. scabiei* possess this protein. Therefore Sar s 14 has been proposed to be utilised in ELISA tests diagnosing scabies mite infestation in all host species. We confirmed high amino-acid similarity between *S. scabiei* var *suis* and *var. hominis* Sar s 14.3, and demonstrated that sera from infected pigs recognised the *S. scabiei* var. *hominis* derived recombinant protein. When considering the suitability of Sar s 14.3 for diagnosis of mange in other animals, or if using different recombinant proteins, it is important to assess potential genetic differences between different host derived mites. With the availability of whole genome sequences possible in the future, this task will become more straightforward.

ELISA based diagnosis using both WMA and Sar s 14.3 was highly sensitive and specific for diagnosis of sarcoptic mange from weeks 8–16 post-infection. A key difference between the antigens was that Sar s 14.3 had higher sensitivity than WMA earlier in infection, while WMA was more sensitive in late infection, but is also known to persist in animals for some time after treatment. Decreased sensitivity of *S. scabiei* WMA ELISA has been reported in adult sows compared to piglets (50% compared to 80%) [65], so age related factors or adaptive immunity may play a role in our observations of declining Sar s 14.3. The fact that pigs were still clinically infected at weeks 20 and 24, but antibodies to Sar s 14.3 had declined to normal by this time, may be a limitation for diagnostic usefulness in long term or chronic infections. To investigate this important issue in more detail, larger studies on adult pigs need to be undertaken. It is also possible that a larger protein fragment or fragments from Sar s 14, or a cocktail of recombinant proteins may further improve sensitivity for long term infections. Although it is important for an ELISA to detect current infection with sufficient sensitivity, the lack of a prolonged response to Sar s 14.3 could be considered an advantage if the objective is to differentiate recent from not recent infestation. While similar temporal studies in humans are not ethically acceptable, monitoring the kinetics of antibody responses after treatment and reinfection in both humans, and in our pig model, would be of value.

### Conclusion

In the absence of the availability of host specific whole mite extracts for use in serodiagnostics, recombinant proteins represent a sustainable option. Results from this study indicate that the *S. scabiei* antigen Sar s 14.3 provides high sensitivity for recent clinical sarcoptic mange infection. Although further assay optimisation may be needed, we have now established that Sar s 14.3 may be a promising tool for diagnosis of mange in both humans and pigs.

## Supporting Information

Figure S1 **Comparison of IgG ELISA utilising IgG depleted and untreated mite extracts.** To remove potential contaminating host IgG from whole mite extracts, protein extracts were passed through an IgG depletion column. Equal amounts of depleted and non-depleted WMA extracts were used to coat wells and total IgG ELISA done with pooled mange positive and negative control sera using standard protocols. Results show mean ±SEM from two experiments. No significant differences in binding were observed between extracts (p = 0.94, students T-test).
(TIF)

Figure S2 **Testing of pre-trial parent sows by Sar s 14.3 IgG ELISA.** Grey bars represent individual parent sows, Mange infected (black bars) and non-infected (white bars) adult pigs were used as positive and negative controls. Bars represent mean +SEM.
(TIFF)
