# Supplementary figures and images for: Antibody Responses to Sarcoptes scabiei Apolipoprotein in a Porcine Model: Relevance to Immunodiagnosis of Recent Infection
Source: PLoS One. 2013 Jun 6;8(6):e65354. doi: 10.1371/journal.pone.0065354 (PMC3675102; doi:10.1371/journal.pone.0065354)

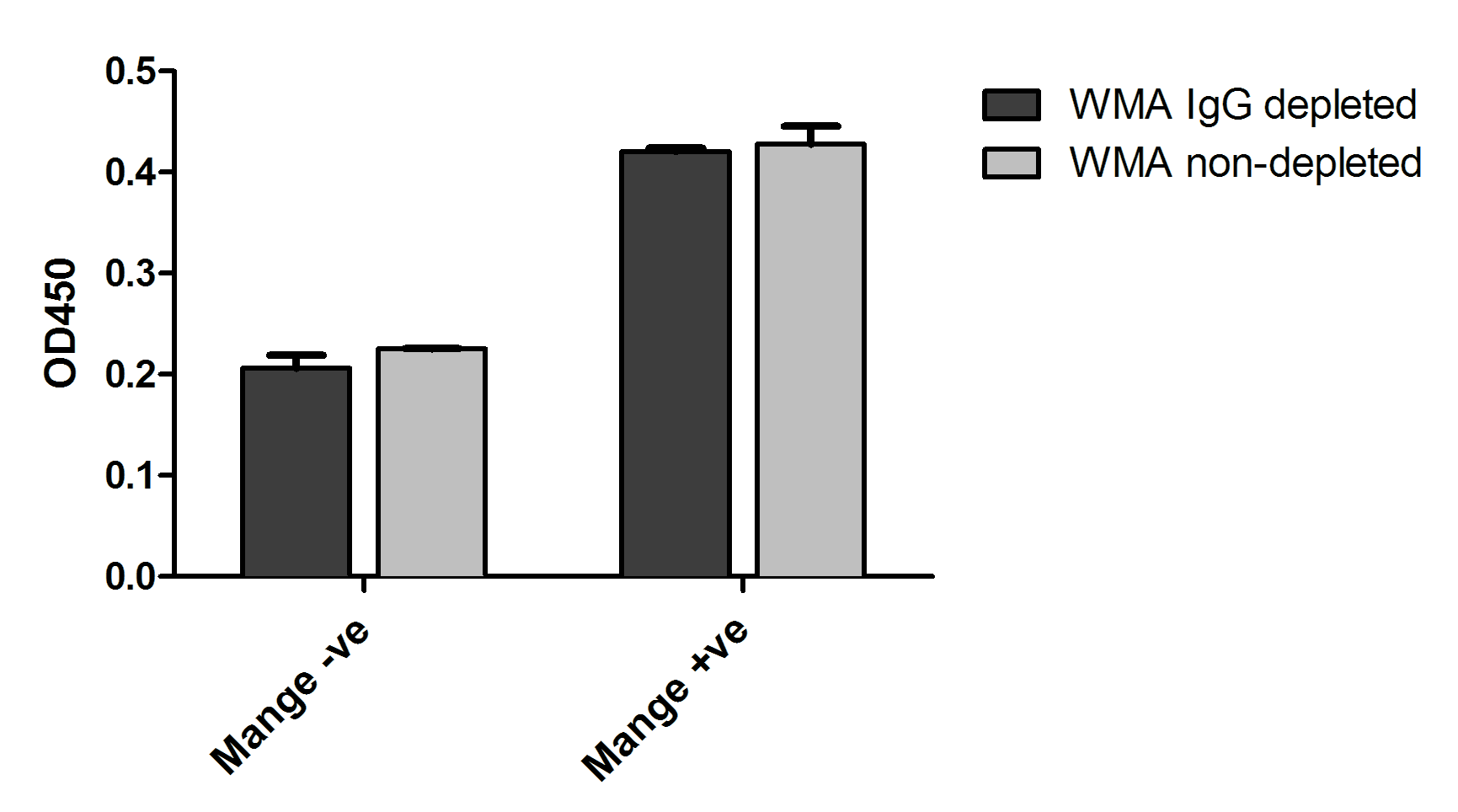

Supplement: Figure S1 — Comparison of IgG ELISA utilising IgG depleted and untreated mite extracts. To remove potential contaminating host IgG from whole mite extracts, protein extracts were passed through an IgG depletion column. Equal amounts of depleted and non-depleted WMA extracts were used to coat wells and total IgG ELISA done with pooled mange positive and negative control sera using standard protocols. Results show mean ±SEM from two experiments. No significant differences in binding were observed between extracts (p = 0.94, students T-test). (TIF) [file pone.0065354.s001.tif]

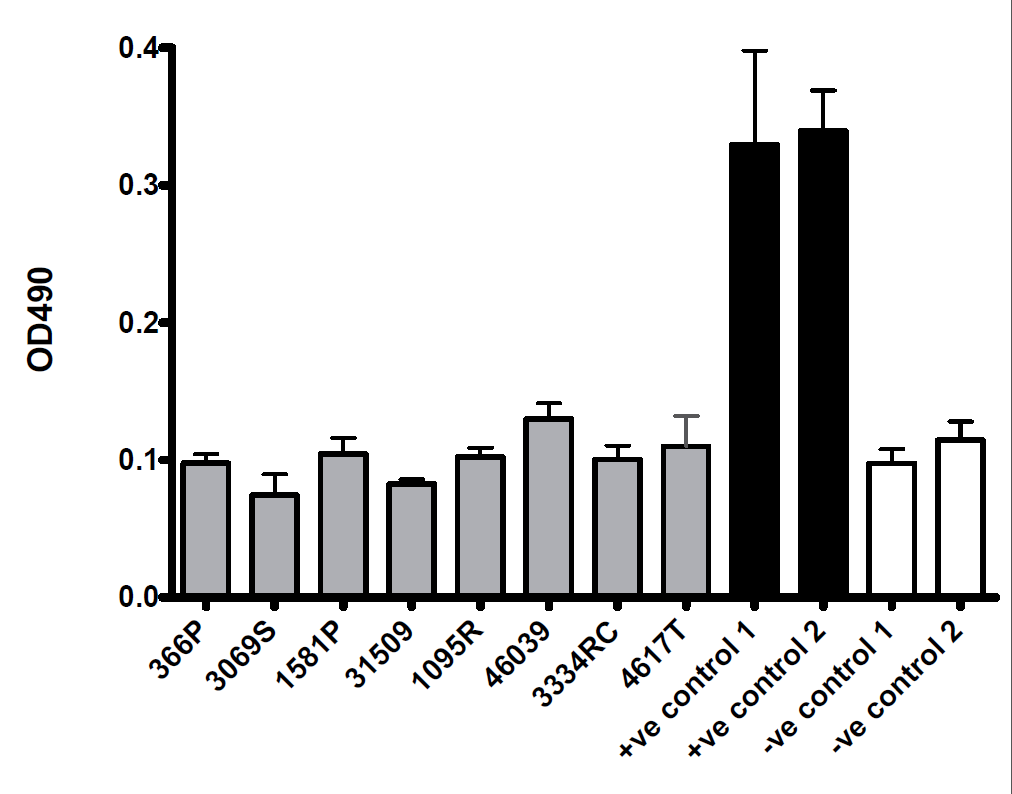

Supplement: Figure S2 — Testing of pre-trial parent sows by Sar s 14.3 IgG ELISA. Grey bars represent individual parent sows, Mange infected (black bars) and non-infected (white bars) adult pigs were used as positive and negative controls. Bars represent mean +SEM. (TIFF) [file pone.0065354.s002.tiff]
